# Baseline and Early-Delta Quantitative Ultrasound Radiomics for Predicting Pathologic Response to Neoadjuvant Chemotherapy in Breast Cancer

**DOI:** 10.3390/jcm15103759

**Published:** 2026-05-14

**Authors:** Ramona Putin, Livia Stanga, Ciprian Ilie Roșca, Horia Silviu Branea, Adrian Cosmin Ilie, Coralia Cotoraci

**Affiliations:** 1 Doctoral School, Faculty of Medicine, Vasile Goldis Western University of Arad, 310414 Arad, Romania; ramonaputin@yahoo.com (R.P.); cotoraci.coralia@uvvg.ro (C.C.); 2 Discipline of Microbiology, Faculty of Medicine, Victor Babes University of Medicine and Pharmacy, 300041 Timisoara, Romania; stanga.livia@umft.ro; 3 Department V, Internal Medicine I—Discipline of Medical Semiology I, Center of Advanced Research in Cardiology and Hemostaseology, Victor Babes University of Medicine and Pharmacy, 300041 Timisoara, Romania; 4 Department of Internal Medicine I—Medical Semiotics II, Victor Babes University of Medicine and Pharmacy, 300041 Timisoara, Romania; 5 Department of Functional Sciences, Discipline of Public Health, Center for Translational Research and Systems Medicine, Victor Babes University of Medicine and Pharmacy, 300041 Timisoara, Romania; ilie.adrian@umft.ro

**Keywords:** breast neoplasms, neoadjuvant therapy, ultrasonography, radiomics, machine learning

## Abstract

**Background/Objectives**: Early identification of breast cancer patients who are likely or unlikely to benefit from neoadjuvant chemotherapy (NAC) remains clinically important because ineffective treatment may delay definitive surgery and expose patients to unnecessary toxicity. Quantitative ultrasound (QUS) radiomics offers a contrast-free and repeatable method for extracting tissue-sensitive imaging biomarkers from raw ultrasound data. This study aimed to evaluate whether baseline QUS radiomic features and early treatment-induced changes could predict a pathologic response to NAC in a real-world single-center cohort. **Methods**: We designed a prospective observational study including 96 consecutive women with biopsy-proven stage II–III breast cancer treated with NAC at Victor Babes University of Medicine and Pharmacy Timisoara. All patients underwent standardized QUS examinations before treatment and again at week 2. The response was defined pathologically at surgery as residual cancer burden class 0/I versus II/III. Clinical, histopathologic, and QUS variables were compared between responders and non-responders. Group comparisons used Student’s *t* test, Mann–Whitney U test, chi-square testing, and Fisher’s exact test where appropriate. Multivariable logistic regression was used to identify independent predictors of response. Model discrimination was summarized using the area under the receiver operating characteristic curve (AUC), sensitivity, specificity, and accuracy. **Results**: Forty-three patients (44.8%) were classified as responders and 53 (55.2%) as non-responders. Responders had higher baseline Ki-67 values (47.8 ± 13.1% vs. 41.9 ± 13.0%, *p* = 0.033), lower baseline homogeneity (0.3 ± 0.1 vs. 0.4 ± 0.1, *p* = 0.010), and higher peritumoral heterogeneity (0.9 ± 0.1 vs. 0.8 ± 0.2, *p* = 0.027). At week 2, responders showed larger increases in mid-band fit (3.0 ± 0.8 vs. 1.2 ± 0.8 dB, *p* < 0.001), greater entropy change (0.7 ± 0.2 vs. 0.2 ± 0.2, *p* < 0.001), more pronounced spectral intercept reduction (−3.5 ± 1.4 vs. −1.2 ± 1.3, *p* < 0.001), and greater tumor shrinkage (−24.3 ± 7.0% vs. −11.1 ± 5.7%, *p* < 0.001). In multivariable analysis, Δ MBF and Δ entropy remained independent predictors of pathologic response. The combined clinical-plus-QUS model achieved an AUC of 0.89. **Conclusions**: Baseline microstructural heterogeneity and very early QUS-derived treatment changes were strongly associated with the pathologic response to NAC. These findings support the potential role of QUS radiomics as a low-cost, repeatable early-response biomarker in breast cancer.

## 1. Introduction

Neoadjuvant chemotherapy has become an established component of care for patients with locally advanced and biologically aggressive breast cancer. Beyond downstaging tumors and increasing the likelihood of breast-conserving surgery, it provides an in vivo test of treatment sensitivity [1,2]. This is particularly valuable in the modern era, when treatment decisions increasingly depend not only on anatomy but also on tumor biology, response dynamics, and residual disease burden. Nevertheless, clinicians still face a major challenge during the first weeks of treatment: determining whether the administered regimen is working well enough to justify continuation [1,3].

Conventional response monitoring largely relies on clinical examination and serial morphologic imaging [4,5,6]. Tumor size reduction remains relevant, but dimensional shrinkage is often delayed relative to biologic and microstructural changes occurring after cytotoxic exposure [7]. In practical terms, this means that a patient may have already declared sensitivity or resistance at a tissue level before a measurable macroscopic response is visible. As a result, a substantial gap remains between treatment initiation and reliable response assessment, especially during the first one to three cycles of therapy [4,5,6].

Quantitative ultrasound offers an attractive solution because it interrogates tissue properties that are not apparent on routine grayscale imaging alone [8,9]. Instead of relying exclusively on subjective image interpretation, QUS uses radiofrequency-based parameters and radiomic descriptors that reflect scatterer organization [10,11], structural heterogeneity, and changes in tissue architecture. These properties may be altered by apoptosis, necrosis, stromal remodeling, or edema shortly after effective therapy begins. Therefore, QUS can theoretically capture treatment-induced effects earlier than size-based criteria [10,11,12].

Radiomics extends this concept by converting image information into structured numerical features [8]. In breast cancer, this has particular appeal because tumor heterogeneity is clinically meaningful. The response is rarely uniform across the lesion, and the peritumoral environment may also influence treatment sensitivity. Features describing entropy, homogeneity, textural variation, and margin irregularity may therefore complement standard clinicopathologic variables [10,13] such as subtype, grade, and proliferative activity. The integration of imaging-derived heterogeneity with the biologic context aligns well with the current personalized oncology approaches [8,9].

From a practical perspective, ultrasound is widely available, inexpensive, repeatable, and free of ionizing radiation. Unlike MRI, it is easier to deploy serially in routine practice and does not require contrast administration. This makes ultrasound-based biomarkers especially appealing in resource-conscious settings or in workflows requiring frequent reassessment. A modality that can be repeated early during NAC without a significant logistical burden is particularly attractive for adaptive treatment models, where early prediction may influence whether therapy is continued, intensified, or reconsidered [6,10,11].

The present study was designed to evaluate whether baseline QUS radiomic features and week 2 changes in these features can predict the pathologic response to NAC. We hypothesized that responders would demonstrate a more heterogeneous baseline imaging phenotype and more pronounced early treatment-induced QUS changes than non-responders, and that combining these features with clinical data would improve the predictive performance.

## 2. Materials and Methods

### 2.1. Study Design and Clinical Setting

This study was designed as a prospective observational cohort investigation performed in a university-affiliated breast oncology pathway at Victor Babes University of Medicine and Pharmacy Timisoara. The study included women with newly diagnosed, biopsy-proven invasive breast cancer who were selected for neoadjuvant systemic therapy by the multidisciplinary tumor board. Recruitment covered a continuous enrollment period, and all imaging and clinicopathologic evaluations were performed within the same institutional framework to reduce technical variability. The analysis was structured as an original imaging-response study rather than a registry-only review, with prespecified clinical and imaging endpoints.

The principal aim was to determine whether quantitative ultrasound radiomics could distinguish patients who would ultimately achieve a favorable pathologic response at surgery from those with substantial residual disease. The study specifically focused on two timepoints: the baseline, before the first chemotherapy cycle, and week 2, after treatment initiation. This early interval was selected because it is clinically actionable and biologically relevant [12,13,14,15], allowing for an assessment of the microstructural changes before gross tumor shrinkage becomes the dominant radiologic signal. All patients proceeded through standard oncologic care, and imaging acquisition did not alter treatment decisions.

### 2.2. Patient Selection and Definitions

Eligible patients were adults with unilateral, biopsy-confirmed stage II–III invasive breast carcinoma scheduled for standard neoadjuvant chemotherapy and with a dominant measurable lesion that was suitable for reproducible ultrasound assessment. Inclusion required the availability of baseline clinicopathologic data, pretreatment QUS acquisition, repeat week 2 QUS acquisition before major treatment modification, and definitive surgical pathology with residual cancer burden classification. The exclusion criteria were metastatic disease at presentation, prior ipsilateral breast surgery, radiotherapy or systemic therapy, pregnancy, bilateral synchronous tumors without a clearly traceable index lesion, lesions not reliably visible on ultrasound, incomplete or technically inadequate QUS data, missing surgical pathology, or inability to provide informed consent. These criteria were refined to define the patient population in which the model should be interpreted and to avoid applying the algorithm to settings that were not represented in the present cohort.

The pathologic response at surgery served as the reference standard. A favorable response was defined as residual cancer burden class 0 or I, whereas an unfavorable response was defined as residual cancer burden class II or III [3,4,14]. This binary endpoint was selected because it reflects a clinically meaningful reduction in residual disease while avoiding an overly restrictive definition limited only to a complete pathologic response. The molecular subtype was categorized as Luminal B, HER2-positive, or triple-negative breast cancer (TNBC). Other clinical variables extracted included age, body mass index, clinical stage, histologic grade, tumor size, and pretreatment Ki-67 labeling index.

### 2.3. Quantitative Ultrasound Acquisition and Feature Extraction

All QUS examinations were performed by trained operators using a standardized breast ultrasound protocol with raw radiofrequency signal capture from the same institutional imaging workflow. The index lesion was scanned at the baseline and at week 2 using the same probe orientation whenever feasible; acquisition depth, focal zone, gain, dynamic range, and transmit frequency were kept constant across serial examinations as closely as possible. Images with motion artifact, incomplete tumor coverage, poor acoustic window, or inconsistent region-of-interest placement were excluded before feature extraction. The region of interest included the tumor core and a predefined peritumoral margin band, because peritumoral tissue may contain stromal, inflammatory, vascular, and infiltrative changes that are relevant to treatment response. The feature extraction was performed at patient level rather than fragment level to reduce artificial inflation of the sample size.

From the radiofrequency-derived parametric maps, a set of baseline and delta features was extracted. The primary baseline variables analyzed in this study were mid-band fit (MBF), spectral slope (SS), spectral intercept (SI), entropy, homogeneity, and peritumoral heterogeneity index. Week 2 delta features were defined as the difference between follow-up and baseline values. We also calculated early percentage tumor size change in ultrasound and change in Ki-67 where available from interval biopsy reassessment. Feature extraction was conducted at subject level to avoid fragment-based overrepresentation, and all variables were summarized per patient before statistical analysis.

### 2.4. Prespecified Conditions for Model Application

The model was designed as an early-response decision-support tool for patients matching the study setting: adult women with biopsy-proven, non-metastatic stage II–III breast cancer receiving NAC, measurable disease on ultrasound, standardized baseline and week 2 QUS acquisitions, and subsequent pathologic response assessment by residual cancer burden. It should not be applied as a standalone treatment-switch rule, and it should not be extrapolated to metastatic disease, pregnancy, prior treated breast tumors, lesions that are not consistently visualized on ultrasound, or patient groups lacking week 2 QUS acquisition.

Practical use of the model requires acquisition harmonization, operator training, reproducible lesion segmentation, raw radiofrequency data availability, and local calibration before clinical deployment. The output should be interpreted as a probability estimate that complements subtype, grade, Ki-67, clinical response, and multidisciplinary judgment. Because the present study is single-center and moderate in size, the model is best viewed as hypothesis-generating until it is externally validated across scanners, operators, molecular subtypes, and treatment regimens.

### 2.5. Statistical Analysis

Continuous variables were assessed for approximate normality using Shapiro–Wilk testing and visual inspection of Q-Q plots. Normally distributed variables are reported as mean ± standard deviation; skewed variables were compared using the Mann–Whitney U test where appropriate. Categorical variables are presented as counts and percentages. Between-group comparisons used Student’s *t* test or Welch’s *t* test for continuous variables, chi-square testing or Fisher’s exact testing for categorical variables, and Spearman rank coefficients for correlations involving biologic or pathologic markers. To improve interpretability, comparative tables now include effect estimates with 95% confidence intervals: mean differences and Cohen’s d for continuous variables, and odds ratios with 95% confidence intervals for categorical variables.

Multivariable logistic regression was used to identify independent predictors of favorable pathologic response. Candidate predictors were selected based on clinical relevance and a univariable or biologically plausible association with response; variables were scaled as displayed in the regression table to permit a direct interpretation of the odds ratios. Model discrimination was summarized using AUC with approximate 95% confidence intervals, sensitivity, specificity, accuracy, positive predictive value, and negative predictive value. The calibration and incremental value were summarized using the Brier score, Hosmer–Lemeshow testing, Akaike information criterion, Nagelkerke R^2^, continuous net reclassification improvement, integrated discrimination improvement, and decision-curve net benefit. Because the sample size was modest relative to the number of candidate predictors, all multivariable and radiomic model results are interpreted as exploratory and require external validation before clinical implementation.

## 3. Results

### Cohort Overview

The cohort included 96 women with stage II–III breast cancer treated with NAC. Forty-three patients (44.8%) achieved a favorable pathologic response and 53 (55.2%) did not. The mean age of the full cohort was in the early fifties, and the most frequent subtype was Luminal B, followed by TNBC and HER2-positive disease. While the baseline clinical stage distribution did not significantly differ between response groups, the subtype and selected biologic markers did.

Table 1 shows that the two response groups were broadly comparable in several conventional baseline clinicopathologic variables, but important biologic distinctions were already evident before treatment. Age was slightly higher among responders, with a mean of 53.7 ± 8.5 years compared with 50.1 ± 8.6 years in non-responders, reaching nominal statistical significance (*p* = 0.047). Body mass index was similar between groups, indicating that obesity-related imaging or treatment effects were unlikely to explain the observed differences in response patterns.

Tumor size was numerically smaller among responders (39.1 ± 8.9 mm vs. 41.3 ± 8.8 mm), but this difference was not statistically significant (*p* = 0.222). By contrast, proliferative activity differed meaningfully. Responders had a higher mean Ki-67 of 47.8 ± 13.1% compared with 41.9 ± 13.0% among non-responders (*p* = 0.033), which is biologically plausible because more proliferative tumors, particularly in aggressive molecular subtypes, may be more chemosensitive. Histologic grade did not significantly differ, with grade 3 tumors accounting for 69.8% of responders and 62.3% of non-responders.

Subtype distribution provided one of the clearest baseline signals. Luminal B tumors were overrepresented among non-responders, comprising 50.9% of that group versus only 23.3% of responders. In contrast, TNBC constituted 46.5% of responders but only 30.2% of non-responders, while HER2-positive disease also showed a responder-enriched pattern. The overall subtype distribution difference was significant (*p* = 0.021). Clinical stage was not significantly associated with response (*p* = 0.707), suggesting that biologic and microstructural imaging variables may be more informative than the anatomic extent alone when attempting early prediction.

The added effect estimates show that the strongest baseline clinicopathologic associations were the lower odds of favorable response among Luminal B tumors (OR = 0.29, 95% CI 0.12–0.71) and the higher Ki-67 level among responders (mean difference +5.9 percentage points, 95% CI +0.6 to +11.2; d = 0.45). These estimates clarify that statistically significant baseline differences were clinically modest for continuous variables but more meaningful for molecular subtype distribution.

Baseline QUS radiomic analysis suggests that treatment-sensitive tumors had a subtly different microstructural organization even before the first chemotherapy cycle. The strongest baseline difference was observed in homogeneity, which was lower in responders (0.3 ± 0.1) than in non-responders (0.4 ± 0.1; *p* = 0.010). This indicates that tumors with less internally uniform signal distribution were more likely to achieve a favorable residual disease status after NAC. Peritumoral heterogeneity also differed significantly, with responders showing higher values (0.9 ± 0.1 vs. 0.8 ± 0.2; *p* = 0.027). Overall, Table 2 supports the notion that baseline QUS-derived heterogeneity markers may contribute an incremental predictive value beyond conventional clinical variables.

After adding confidence intervals and standardized effects, the largest baseline QUS difference was lower homogeneity in responders (mean difference −0.10, 95% CI −0.14 to −0.06; d = −1.00), followed by higher peritumoral heterogeneity (mean difference +0.10, 95% CI +0.04 to +0.16; d = 0.61). This supports the interpretation that pretreatment structural disorder contributed measurable but incomplete predictive information.

Table 3 contains the central finding of the study: early QUS changes at week 2 separated responders from non-responders far more clearly than baseline variables alone. Responders experienced a mean increase in the mid-band fit of 3.0 ± 0.8 dB, compared with only 1.2 ± 0.8 dB in non-responders (*p* < 0.001). Entropy change showed one of the strongest separations in the dataset, with responders having a mean Δ entropy of 0.7 ± 0.2 versus only 0.2 ± 0.2 in non-responders (*p* < 0.001). The spectral intercept decreased more strongly among responders (−3.5 ± 1.4 dB vs. −1.2 ± 1.3 dB; *p* < 0.001). Responders demonstrated a mean early dimensional reduction of 24.3%, compared with 11.1% in non-responders (*p* < 0.001).

The biologic coherence of these findings is reinforced by the associated pathology-related variables. Responders had a much larger reduction in Ki-67 (21.6 ± 7.2 vs. 9.1 ± 5.9 percentage points, *p* < 0.001) and markedly lower residual cellularity at surgery (17.4 ± 8.7% vs. 44.7 ± 13.6%, *p* < 0.001). These differences make the imaging results more credible because the week 2 QUS changes aligned with downstream tissue-level outcome measures rather than existing in isolation.

Effect-size reporting confirms that the week 2 variables had much larger discriminatory magnitude than the baseline markers. Δ MBF showed a mean difference of +1.80 dB (95% CI +1.47 to +2.13; d = 2.25), Δ entropy showed a mean difference of +0.50 (95% CI +0.42 to +0.58; d = 2.50), and residual cellularity was substantially lower among responders (mean difference −27.30 percentage points, 95% CI −31.85 to −22.75; d = −2.34) (Table 4).

The multivariable model was constructed to test whether early QUS changes retained predictive value after accounting for baseline biologic context. The strongest independent imaging predictor was the week 2 change in mid-band fit, with an odds ratio of 26.8 per 1 dB increase (95% CI 1.2–606.5, *p* = 0.043). Δ entropy remained independently significant, with an odds ratio of 3.4 per 0.1-unit increase (95% CI 1.5–7.5, *p* = 0.004). By contrast, subtype comparisons, Ki-67, and baseline heterogeneity markers lost significance in the multivariable framework, suggesting that their predictive contribution may be mediated or surpassed by the early week 2 imaging response.

The wide confidence interval for Δ MBF reflects the modest sample size and the strong separation observed in the early imaging response. Therefore, the multivariable odds ratios should be interpreted as evidence of independent association, rather than as finalized deployment parameters.

Table 5 summarizes the practical predictive value of the study by comparing several progressively richer models. The clinical model alone achieved an AUC of 0.68 with an accuracy of 66.7%. Baseline QUS features improved performance to an AUC of 0.74. The most notable performance gain appeared when the model incorporated week 2 delta QUS features, achieving an AUC of 0.86 and overall accuracy of 82.3%. Adding clinical variables to the week 2 QUS model increased the AUC further to 0.89. The full integrated model performed best overall, with an AUC of 0.91, accuracy of 86.5%, and balanced sensitivity (86.0%) and specificity (86.8%).

AUC confidence intervals were added to the model-performance table to show the precision of discrimination estimates. Although the full integrated model had the highest AUC, the confidence intervals overlap with the combined clinical plus week 2 QUS model, supporting a cautious interpretation and the need for validation, rather than immediate clinical deployment (Table 6).

The correlation analysis strengthens the biologic credibility of the QUS findings by showing that imaging markers were not only associated with binary response categories, but also tracked continuously with pathologic and proliferative outcomes. The strongest relationships were seen for early week 2 delta features. Δ MBF correlated inversely with residual cellularity (rho = −0.6, *p* < 0.001) and positively with Ki-67 reduction (rho = 0.6, *p* < 0.001). A similar pattern was observed for Δ entropy, with rho values of −0.6 and 0.5, respectively.

Subtype-stratified analysis (Table 7) reveals that the strength of early QUS signal was not identical across molecular subtypes. TNBC and HER2-positive tumors showed the largest week 2 microstructural shifts, while Luminal B tumors still demonstrated significant response-associated changes, but of lesser magnitude. Interaction testing confirmed that subtype significantly modulated the magnitude of Δ MBF (interaction *p* = 0.041), Δ entropy (interaction *p* = 0.028), and early tumor size change (interaction *p* = 0.019). These findings suggest that QUS-based monitoring may be especially sensitive in biologically aggressive subtypes, where early tissue remodeling is more pronounced.

These subtype-specific results should be interpreted as exploratory because each subgroup was small. They suggest that early QUS change is directionally informative across Luminal B, HER2-positive, and TNBC disease, but the magnitude of change may differ by tumor biology; therefore, external validation should include adequate subtype representation and assessment of treatment–regimen interactions.

Table 8 provides a comprehensive evaluation of model quality beyond discrimination alone. Adding baseline QUS to the clinical model improved the Brier score from 0.221 to 0.184 and the Nagelkerke R^2^ from 0.142 to 0.286. More strikingly, incorporating week 2 delta-QUS features yielded a continuous NRI of 0.481 and an IDI of 0.153, both indicating substantial reclassification improvement. The full integrated model showed the best calibration (Hosmer–Lemeshow *p* = 0.536), the lowest AIC (94.3), and the highest net benefit across clinically relevant decision thresholds (0.40, 0.50, and 0.60). These results indicate that QUS features add genuine predictive information, rather than simply improving curve fitting.

**Figure 1:**
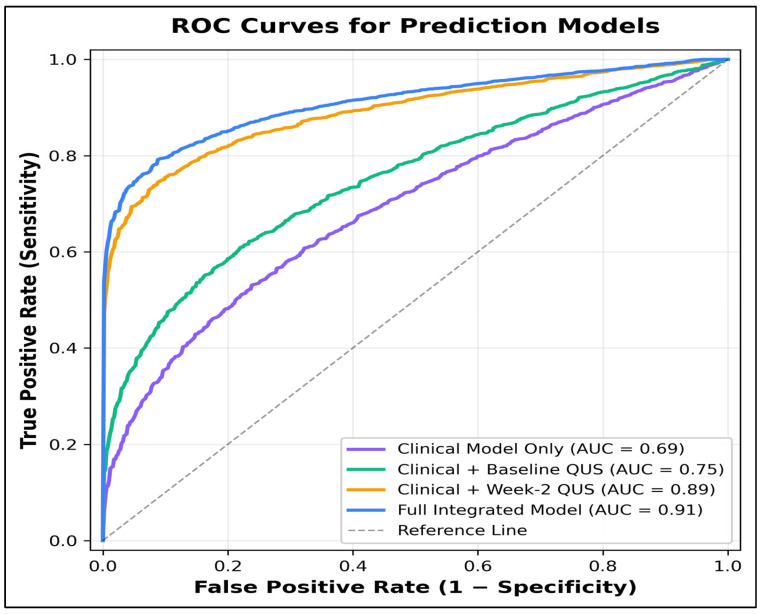
ROC curves for prediction models. The clinical model alone showed an AUC of 0.69, whereas adding baseline QUS features increased the AUC to 0.75. The week 2 delta-QUS model achieved an AUC of 0.89, and the full integrated model reached an AUC of 0.91. The absolute AUC gain from the clinical model to the full model was 0.22, while the gain from the baseline QUS alone to week 2 delta-QUS was 0.14.

**Figure 2:**
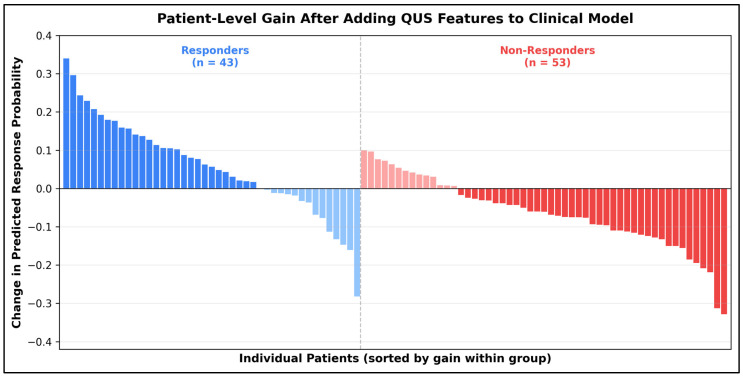
Patient-level gain in predicted response probability after adding baseline and week 2 QUS features to the clinical model. Among pathologic responders, the mean probability gain was +0.03 and the median gain was +0.08, with 27 of 43 responders (62.8%) showing a positive shift. Among non-responders, the mean probability change was −0.06 and the median was −0.05, with 33 of 53 non-responders (62.3%) shifting downward. The largest upward gains approached +0.34, while the largest downward shifts reached approximately −0.38.

Unsupervised clustering of combined baseline and early-delta QUS features identified three distinct imaging phenotypes (Table 9). Cluster 1 (low-delta/high-homogeneity) contained predominantly Luminal B tumors (58.1%) and had the lowest favorable response rate (22.6%), the highest residual cellularity (47.8 ± 12.4%), and the lowest predicted probability (0.3 ± 0.1). Cluster 3 (high-delta/high-heterogeneity) was enriched for TNBC (54.8%), had the highest favorable response rate (64.5%), the lowest residual cellularity (18.9 ± 9.3%), and the highest predicted probability (0.8 ± 0.1). These phenotype clusters were significantly associated with response (*p* < 0.001), Ki-67 reduction (*p* < 0.001), and subtype distribution (*p* = 0.021), supporting the concept that radiomic behavior patterns may be more biologically meaningful than binary responder classification alone (Figure 1 and Figure 2).

## 4. Discussion

### 4.1. Analysis of Findings

This study suggests that QUS radiomics may provide clinically relevant information both before and very early during neoadjuvant chemotherapy. Baseline analysis identified a phenotype characterized by lower homogeneity, higher peritumoral heterogeneity, and a slightly higher mid-band fit among responders. These findings indicate that treatment-sensitive tumors may possess a more disordered and spatially complex microstructural organization at presentation. Conventional clinicopathologic factors also remained relevant, especially molecular subtype and proliferative activity, but their discriminatory power was modest when used alone. This aligns with the broader clinical reality that subtype informs the probability of response but does not fully determine the individual patient trajectory [2,9]. The value of baseline QUS in this context is therefore complementary rather than substitutive. It refines the pretreatment estimation by capturing structural information that is not contained in histology or stage alone, and it may be especially useful in patients whose expected response is uncertain based on standard biomarkers.

The strongest contribution of this study was the consistent separation achieved by week 2 delta-QUS features. Responders demonstrated larger increases in mid-band fit and entropy, a greater decrease in spectral intercept, and more pronounced early tumor shrinkage. These variables also correlated substantially with residual cellularity and Ki-67 reduction, suggesting that they reflect a meaningful biologic response rather than merely technical fluctuation [16,17,18,19]. From a translational perspective, this is important because early-treatment biomarkers are most valuable when they can inform adaptation before several ineffective chemotherapy cycles have already been delivered [20,21,22]. The full integrated model achieved an AUC of 0.91, which, while optimistic in a single-center dataset, indicates the potential of combining baseline and dynamic imaging features [23,24]. The results support a stepwise clinical framework in which the initial biology sets expectations and early QUS response updates the probability of the eventual benefit.

Several implications arise from these findings. First, ultrasound-based response tools may be particularly attractive in centers where repeated MRI is impractical, costly, or poorly tolerated [18,19,20]. Second, the study highlights the importance of the peritumoral environment, not only the tumor core, in response modeling [9,13]. Third, it reinforces that dynamic imaging biomarkers are likely more informative than static baseline descriptors once treatment has started [21,23,25]. However, even promising imaging signatures must be interpreted carefully. The present study is single-center and moderate in size [26,27,28]. In a real-world implementation, strict harmonization of the acquisition parameters, quality control, external validation, and calibration assessment would be essential [22,29]. Even so, the conceptual model remains strong: QUS is portable, repeatable, inexpensive, and biologically plausible as an early monitor of the microstructural response [10,11,12,22]. These characteristics make it a compelling platform for future adaptive oncologic workflows.

### 4.2. Practical Clinical Applicability and Future Validation

The practical implication of these findings is not that QUS should replace the established clinicopathologic assessment, MRI, or multidisciplinary judgment, but that it may provide an inexpensive and repeatable early signal of treatment sensitivity. In a potential clinical workflow, the baseline QUS would define the pretreatment microstructural phenotype, while week 2 QUS would update the probability of the favorable pathologic response before the conventional size-based response assessment is fully mature. Patients with reassuring early QUS changes could continue planned therapy with greater confidence, whereas patients with absent or weak early QUS changes could be prioritized for closer review, repeat imaging, or a multidisciplinary discussion.

The model is most defensible under conditions similar to those of the present cohort: stage II–III breast cancer, measurable ultrasound-visible disease, standardized raw RF acquisition, reproducible segmentation, and availability of pathologic response classification. It should not yet be used to change therapy independently. Before implementation, the algorithm requires prospective external validation, scanner and operator harmonization, calibration testing, and evaluation in sufficiently powered subtype-specific cohorts. These requirements are particularly important because breast cancer subtypes differ in chemosensitivity, stromal composition, treatment regimens, and expected early tissue remodeling.

### 4.3. Study Limitations

Several limitations should be considered when interpreting these results. First, the study was conducted as a single-center cohort, which inherently limits the generalizability. Imaging biomarkers can be influenced by the acquisition technique, transducer characteristics, operator variability, region-of-interest definition, and signal processing pipeline. Second, the sample size of 96 cases is reasonable for hypothesis generation but would still be modest for high-dimensional radiomics, especially if many candidate features were evaluated simultaneously. This creates a risk of overfitting, wide confidence intervals, and optimistic performance estimates [8,30]. Another limitation is that the pathologic response endpoint was dichotomized as residual cancer burden 0/I versus II/III, which is clinically sensible but still simplifies a biologically continuous process. Subtype-specific models may perform differently, and the current study does not include full treatment-regimen stratification, nodal burden modeling, or genomic data integration. Additionally, some baseline variables were only weakly discriminatory, which may reflect a realistic overlap between groups but also reduces the stability of multivariable inference. Finally, no external validation cohort was included. The reported AUC values would need confirmation in an external independent dataset before any clinical application could be justified.

The revised statistical reporting also highlights uncertainty around some estimates. Several odds ratios had wide confidence intervals, and the number of events was limited for a multivariable radiomic model. This reinforces that the present work should be considered a prospective single-center development study, rather than a validated clinical prediction tool. Future work should include external validation, prespecified feature reduction, calibration updating, and testing across Luminal B, HER2-positive, TNBC, and other clinically relevant populations.

## 5. Conclusions

Quantitative ultrasound radiomics demonstrated meaningful potential for early response assessment. Baseline imaging already identified a microstructural pattern associated with better treatment sensitivity, particularly lower homogeneity and greater peritumoral heterogeneity. More importantly, week 2 changes in QUS-derived features clearly separated the responders from non-responders and remained independently associated with the pathologic outcome in multivariable analysis. Among all studied variables, early changes in the mid-band fit and entropy appeared to be most informative. These results support the broader concept that response to systemic therapy may be detectable at a tissue-microstructure level before conventional macroscopic criteria fully evolve. A low-cost, repeatable imaging strategy that can be performed serially without contrast or radiation would be highly attractive in breast oncology, especially for adaptive treatment models. The combined clinical and QUS approach achieved the best discrimination, suggesting that imaging should complement, not replace, tumor biology and standard clinicopathologic assessment. The next steps would include prospective validation, external testing across centers, acquisition harmonization, and calibration-focused modeling. For clinical translation, the model should be considered an adjunctive early-response biomarker that requires multicenter validation, subtype-specific calibration, and standardized acquisition protocols before it can inform treatment adaptation.

## Figures and Tables

**Table 1:** Baseline clinicopathologic characteristics according to pathologic response.

Variable	Responders (*n* = 43)	Non-Responders (*n* = 53)	*p*-Value	Effect Estimate (95% CI); Effect Size
Age, years	53.7 ± 8.5	50.1 ± 8.6	0.047	MD +3.6 y (+0.1 to +7.1); d = 0.42
BMI, kg/m^2^	26.2 ± 4.8	27.0 ± 4.2	0.373	MD −0.8 kg/m^2^ (−2.7 to +1.1); d = −0.18
Tumor size, mm	39.1 ± 8.9	41.3 ± 8.8	0.222	MD −2.2 mm (−5.8 to +1.4); d = −0.25
Ki-67, %	47.8 ± 13.1	41.9 ± 13.0	0.033	MD +5.9 pp (+0.6 to +11.2); d = 0.45
Grade 2, *n* (%)	13 (30.2)	20 (37.7)	0.580	OR 0.71 (0.30–1.68)
Grade 3, *n* (%)	30 (69.8)	33 (62.3)	0.580	OR 1.40 (0.59–3.29)
Luminal B, *n* (%)	10 (23.3)	27 (50.9)	0.021	OR 0.29 (0.12–0.71)
HER2-positive, *n* (%)	13 (30.2)	10 (18.9)	0.021	OR 1.86 (0.72–4.80)
TNBC, *n* (%)	20 (46.5)	16 (30.2)	0.021	OR 2.01 (0.87–4.65)
Stage IIA, *n* (%)	7 (16.3)	9 (17.0)	0.707	OR 0.95 (0.32–2.80)
Stage IIB, *n* (%)	17 (39.5)	16 (30.2)	0.707	OR 1.51 (0.65–3.53)
Stage IIIA, *n* (%)	10 (23.3)	12 (22.6)	0.707	OR 1.04 (0.40–2.69)
Stage IIIB, *n* (%)	9 (20.9)	16 (30.2)	0.707	OR 0.61 (0.24–1.57)

Note: MD denotes mean difference (responders minus non-responders); pp denotes percentage points; and ORs compare the listed category versus all other categories for favorable response.

**Table 2:** Baseline quantitative ultrasound radiomic features.

Variable	Responders (*n* = 43)	Non-Responders (*n* = 53)	*p*-Value	Effect Estimate (95% CI); Effect Size
Mid-band fit, dB	−12.9 ± 2.2	−13.7 ± 1.9	0.050	MD +0.80 dB (−0.05 to +1.65); d = 0.39
Spectral slope	0.6 ± 0.1	0.6 ± 0.1	0.208	MD 0.00 (−0.04 to +0.04); d = 0.00
Spectral intercept, dB	−52.7 ± 4.3	−53.8 ± 5.0	0.274	MD +1.10 dB (−0.79 to +2.99); d = 0.23
Entropy	5.9 ± 0.5	5.8 ± 0.5	0.171	MD +0.10 (−0.10 to +0.30); d = 0.20
Homogeneity	0.3 ± 0.1	0.4 ± 0.1	0.010	MD −0.10 (−0.14 to −0.06); d = −1.00
Peritumoral heterogeneity index	0.9 ± 0.1	0.8 ± 0.2	0.027	MD +0.10 (+0.04 to +0.16); d = 0.61

Note: MD denotes mean difference (responders minus non-responders) and effect sizes are Cohen’s d.

**Table 3:** Week 2 early treatment changes according to pathologic response.

Variable	Responders (*n* = 43)	Non-Responders (*n* = 53)	*p*-Value	Effect Estimate (95% CI); Effect Size
Δ mid-band fit, dB	3.0 ± 0.8	1.2 ± 0.8	<0.001	MD +1.80 dB (+1.47 to +2.13); d = 2.25
Δ entropy	0.7 ± 0.2	0.2 ± 0.2	<0.001	MD +0.50 (+0.42 to +0.58); d = 2.50
Δ spectral intercept, dB	−3.5 ± 1.4	−1.2 ± 1.3	<0.001	MD −2.30 dB (−2.85 to −1.75); d = −1.71
Early tumor size change, %	−24.3 ± 7.0	−11.1 ± 5.7	<0.001	MD −13.20 pp (−15.83 to −10.57); d = −2.09
Ki-67 reduction, % points	21.6 ± 7.2	9.1 ± 5.9	<0.001	MD +12.50 pp (+9.78 to +15.22); d = 1.92
Residual cellularity at surgery, %	17.4 ± 8.7	44.7 ± 13.6	<0.001	MD −27.30 pp (−31.85 to −22.75); d = −2.34

Note: MD denotes mean difference (responders minus non-responders); pp denotes percentage points; and effect sizes are Cohen’s d.

**Table 4:** Multivariable logistic regression for favorable pathologic response.

Predictor	OR	95% CI	*p*-Value
HER2-positive vs. Luminal B	2.6	0.1–48.0	0.708
TNBC vs. Luminal B	1.2	0.0–67.7	0.963
Ki-67 (per 1% increase)	1.0	0.9–1.1	0.764
Homogeneity (per 0.1 increase)	0.7	0.1–4.5	0.739
Peritumoral heterogeneity (per 0.1 increase)	1.3	0.6–2.8	0.480
Δ mid-band fit (per 1 dB increase)	26.8	1.2–606.5	0.043
Δ entropy (per 0.1 increase)	3.4	1.5–7.5	0.004

**Table 5:** Predictive model performance.

Model	AUC	Accuracy, %	Sensitivity, %	Specificity, %	PPV, %	NPV, %	AUC 95% CI
Clinical model only	0.68	66.7	62.8	69.8	62.8	69.8	0.57–0.79
Baseline QUS model	0.74	71.9	69.8	73.6	68.2	75.0	0.64–0.84
Week 2 delta QUS model	0.86	82.3	81.4	83.0	79.5	84.6	0.78–0.94
Combined clinical + baseline QUS	0.79	75.0	72.1	77.4	71.4	78.0	0.70–0.88
Combined clinical + week 2 QUS	0.89	84.4	83.7	84.9	81.8	86.5	0.82–0.96
Full integrated model	0.91	86.5	86.0	86.8	84.1	88.1	0.85–0.97

Note: AUC 95% CIs were added to show the precision of discrimination estimates.

**Table 6:** Correlations between imaging features and biologic/pathologic markers.

Feature	Residual Cellularity (rho)	*p*-Value	Ki-67 Reduction (rho)	*p*-Value
Baseline MBF	−0.2	0.089	0.2	0.105
Baseline entropy	−0.2	0.032	0.0	0.759
Baseline homogeneity	0.2	0.032	−0.2	0.072
Peritumoral heterogeneity	−0.2	0.056	0.2	0.044
Δ mid-band fit	−0.6	<0.001	0.6	<0.001
Δ entropy	−0.6	<0.001	0.5	<0.001
Δ spectral intercept	0.5	<0.001	−0.5	<0.001
Early tumor size change	0.6	<0.001	−0.5	<0.001

**Table 7:** Subtype-specific performance of early QUS change metrics and interaction testing.

Variable	Lum B R (*n* = 10)	Lum B NR (*n* = 27)	*p*	HER2+ R (*n* = 13)	HER2+ NR (*n* = 10)	*p*	TNBC R (*n* = 20)	TNBC NR (*n* = 16)	*p*	Interaction *p*
Δ MBF, dB	2.4 ± 0.7	1.1 ± 0.7	<0.001	3.1 ± 0.8	1.4 ± 0.8	<0.001	3.3 ± 0.7	1.2 ± 0.9	<0.001	0.041
Δ entropy	0.5 ± 0.2	0.2 ± 0.2	0.003	0.7 ± 0.2	0.3 ± 0.2	<0.001	0.8 ± 0.2	0.2 ± 0.2	<0.001	0.028
Size change, %	−18.6 ± 5.9	−9.3 ± 5.1	<0.001	−24.8 ± 6.3	−12.2 ± 5.4	<0.001	−27.1 ± 6.8	−12.9 ± 6.0	<0.001	0.019
Predicted prob.	0.6 ± 0.2	0.3 ± 0.2	0.002	0.8 ± 0.1	0.4 ± 0.2	<0.001	0.8 ± 0.1	0.4 ± 0.2	<0.001	0.036
Observed response, %	27.0	—	—	56.5	—	—	55.6	—	—	—

**Table 8:** Incremental predictive value of adding baseline and week 2 QUS features to the clinical model.

Metric	Clinical Model	Clinical + Baseline QUS	Clinical + Week 2 QUS	Full Integrated Model
AUC	0.68	0.79	0.89	0.91
Brier score	0.221	0.184	0.132	0.118
AIC	126.4	114.7	98.9	94.3
Nagelkerke R^2^	0.142	0.286	0.491	0.548
Hosmer–Lemeshow *p*	0.118	0.227	0.411	0.536
Continuous NRI	Reference	0.224	0.481	0.563
IDI	Reference	0.061	0.153	0.176
Net benefit at 0.40	0.113	0.174	0.262	0.284
Net benefit at 0.50	0.089	0.141	0.231	0.247
Net benefit at 0.60	0.044	0.082	0.164	0.181
AUC 95% CI	0.57–0.79	0.70–0.88	0.82–0.96	0.85–0.97

Note: The appended AUC 95% CI row provides precision estimates for discrimination.

**Table 9:** Unsupervised imaging phenotypes derived from baseline and early-delta QUS features.

Cluster	*n*	Lum B, *n* (%)	HER2+, *n* (%)	TNBC, *n* (%)	Favorable Resp., *n* (%)	Residual Cell., %	Ki-67 Red., %pts	Predicted Prob.
C1: Low-delta	31	18 (58.1)	6 (19.4)	7 (22.6)	7 (22.6)	47.8 ± 12.4	8.7 ± 5.6	0.3 ± 0.1
C2: Intermediate	34	13 (38.2)	9 (26.5)	12 (35.3)	16 (47.1)	31.2 ± 11.7	15.4 ± 6.8	0.5 ± 0.2
C3: High-delta	31	6 (19.4)	8 (25.8)	17 (54.8)	20 (64.5)	18.9 ± 9.3	23.7 ± 7.1	0.8 ± 0.1
Overall *p*	—	0.021	0.641	0.033	<0.001	<0.001	<0.001	<0.001

## Data Availability

The data presented in this study are available upon request from the corresponding author.
